# The Apocarotenoid Zaxinone Is a Positive Regulator of Strigolactone and Abscisic Acid Biosynthesis in Arabidopsis Roots

**DOI:** 10.3389/fpls.2020.00578

**Published:** 2020-05-14

**Authors:** Abdugaffor Ablazov, Jianing Mi, Muhammad Jamil, Kun-Peng Jia, Jian You Wang, Qitong Feng, Salim Al-Babili

**Affiliations:** ^1^The BioActives Lab, Center for Desert Agriculture, Biological and Environmental Sciences and Engineering, King Abdullah University of Science and Technology, Thuwal, Saudi Arabia; ^2^Institute of Plant Stress Biology, State Key Laboratory of Cotton Biology, Henan University, Kaifeng, China

**Keywords:** carotenoid, apocarotenoid, zaxinone, strigolactone, abscisic acid, phytohormones, growth regulator

## Abstract

Carotenoids are ubiquitous precursors of important metabolites including hormones, such as strigolactones (SLs) and abscisic acid (ABA), and signaling and regulatory molecules, such as the recently discovered zaxinone. Strigolactones and ABA are key regulators of plant growth and development, adaptation to environmental changes and response to biotic and abiotic stress. Previously, we have shown that zaxinone, an apocarotenoid produced in rice by the enzyme zaxinone synthase (ZAS) that is common in mycorrhizal plants, is required for normal rice growth and development, and a negative regulator of SL biosynthesis. Zaxinone is also formed in Arabidopsis, which lacks ZAS, via an unknown route. In the present study, we investigated the biological activity of zaxinone in Arabidopsis, focusing on its effect on SL and ABA biosynthesis. For this purpose, we quantified the content of both hormones and determined the levels of related transcripts in Arabidopsis (*Arabidopsis thaliana*), roots upon zaxinone treatment. For SL quantification, we also employed *Striga* seed germination bioassay. Results obtained show that zaxinone application to hydroponically grown Arabidopsis seedlings enhanced transcript levels of key biosynthetic genes of both hormones, led to higher root ABA and SL (methyl carlactonoate, MeCLA) content, and increased SL release, even under sufficient phosphate supply. Using the SL insensitive (*max2-1*) and the ABA deficient (*aba1-6, aba2-1*, and *nced3*) mutants, we also show that zaxinone application reduced hypocotyl growth and that this effect is caused by increasing ABA content. Our results suggest that zaxinone is a regulatory metabolite also in Arabidopsis, which triggers the biosynthesis of both carotenoid-derived hormones, SLs and ABA, in roots. In the non-mycotrophic plant Arabidopsis, zaxinone does not increase growth and may be perceived as a stress signal, while it acts as a growth-promoting metabolite and suppressor of SL biosynthesis in rice.

## Introduction

Plants produce hormones and chemical signals, which regulate growth, development, and different physiological processes, mediate interactions with surrounding organisms and coordinate plants response to biotic and abiotic stress stimuli (Chaiwanon et al., 2016). Many of these signals originate from secondary metabolic pathways, such as carotenoid biosynthesis (Moise et al., 2013; Sun et al., 2018). Indeed, carotenoids provide precursors for the two important plant hormones abscisic acid (ABA) and strigolactones (SLs) (Cutler et al., 2010; Al-Babili and Bouwmeester, 2015), and for a diverse set of apocarotenoid signaling molecules (Dickinson et al., 2019; Felemban et al., 2019; Fiorilli et al., 2019; Jia et al., 2019). Apocarotenoids are carotenoid derivatives produced through oxidative cleavage of conjugated double bonds in the carotenoid backbone (Nisar et al., 2015; Ahrazem et al., 2016; Jia et al., 2017). Plant apocarotenoids can arise by non-enzymatic oxidation processes that are triggered by ROS, such as singlet oxygen. An example for such apocarotenoids is cyclocitral, a stress signal and root growth regulator (Ramel et al., 2012; Dickinson et al., 2019). However, most of the plants regulatory apocarotenoids are formed by carotenoid cleavage dioxygenases (CCDs) that constitute a ubiquitous, evolutionarily conserved family of non-heme iron (II) dependent enzymes (Giuliano et al., 2003; Nisar et al., 2015; Ahrazem et al., 2016; Jia et al., 2017). The genome of Arabidopsis (*Arabidopsis thaliana*) contains nine CCD genes, including five coding for 9-*cis*-epoxy carotenoid dioxygenases (NCED2, NCED3, NCED5, NCED6, and NCED9) that catalyze the rate-limiting step of ABA biosynthesis, i.e., the cleavage of 9-*cis*-epoxycarotenoids into the ABA precursor xanthoxin and a C_25_-apocarotenoid (Schwartz et al., 1997; Tan et al., 2003; Felemban et al., 2019). The other four genes encode different types of CCDs, which are designated as CCD1, CCD4, CCD7, and CCD8 (Jia et al., 2017). CCD1 enzymes are generally less specific and can convert a wide variety of carotenoid and apocarotenoid substrates, which results in volatile molecules responsible for flavor and aroma in various plant species, and different dialdehyde products (Vogel et al., 2008; Ilg et al., 2009; Ilg et al., 2014). The enzymatic activity of the Arabidopsis CCD4 type determines the carotenoid level in different plant tissues (Gonzalez-Jorge et al., 2013; Bruno et al., 2016; Mi et al., 2019). CCD7 and CCD8 are involved in SL biosynthesis, by catalyzing the sequential conversion of 9-*cis*-β-carotene formed by the isomerase DWARF27 (D27) into the SL biosynthesis intermediate carlactone (CL; Bruno et al., 2014, 2017; Abuauf et al., 2018). In many cases, apocarotenoids are modified by different enzymes, including CCDs themselves or cytochrome P450 enzymes, before acquiring hormone/signaling molecule activity (Zhang et al., 2014; Felemban et al., 2019; Fiorilli et al., 2019). Strigolactones are a novel class of plant hormones best known for their role in determining shoot branching (Gomez-Roldan et al., 2008; Umehara et al., 2008). In addition, SLs regulate a series of further developmental processes (Al-Babili and Bouwmeester, 2015; Waters et al., 2017), secondary growth, and the establishment of root system architecture, and senescence (Al-Babili and Bouwmeester, 2015; Waters et al., 2017; Jia et al., 2019), and are involved in pathogen defense and abiotic stress response (Van Ha et al., 2014; Decker et al., 2017). Besides these hormonal functions, SLs are released through plant roots as intra-specific communication signal that attracts beneficial mycorrhizal fungi for building the arbuscular mycorrhizal (AM) symbiosis (Akiyama et al., 2005). This symbiosis is used by around 80% of land plants to cover their needs for less-accessible minerals, particularly phosphorus, and water (Gutjahr, 2014; Lanfranco et al., 2017). Plants compensate the fungal partner by providing photosynthetic products (Gutjahr, 2014; Lanfranco et al., 2017). Strigolactones induce hyphal branching in AM fungi, which paves the way for establishing the symbiosis and explain why plants produce and release more SLs upon phosphate deficiency (Al-Babili and Bouwmeester, 2015; Waters et al., 2017). However, released SLs are also sensed by seeds of root parasitic weeds, such as *Striga hermonthica*, as a germination signal ensuring the presence of a host needed for their survival as obligate parasites (Xie et al., 2010). Indeed, SLs were originally discovered and identified due to their role in this process (Cook et al., 1966). The biosynthesis of SL starts with all-*trans*/9*-cis-*β-carotene isomerization, which is catalyzed by a *cis/trans* b-carotene isomerase enzyme D27 (Alder et al., 2012; Bruno and Al-Babili, 2016; Abuauf et al., 2018), followed by the MORE AXILLARY GROWTH 3 (MAX3)/CCD7 that cleaves 9-*cis*-β-carotene to produce 9-*cis*-β-apo-10′-carotenal and β-ionone (Alder et al., 2012; Bruno et al., 2014). In the next step, 9-*cis*-β-apo-10′-carotenal is converted by MORE AXILLARY GROWTH 4 (MAX4)/CCD8 that mediates a combination of reactions into CL, the central intermediate of SL biosynthesis (Alder et al., 2012; Bruno et al., 2017; Jia et al., 2017). Carlactone is the substrate of cytochrome P450 enzymes (711 clades), such as the Arabidopsis MORE AXILLARY GROWTH 1 (MAX1), which produce canonical and non-canonical SLs. The Arabidopsis MAX1 oxidizes CL to carlactonoic acid (CLA), which is followed by a methylation step yielding methyl carlactonoate (MeCLA) and a less elucidated lateral branching oxidoreductase (LBO) catalyzed reaction (Supplementary Figure S1; Alder et al., 2012; Challis et al., 2013; Abe et al., 2014; Brewer et al., 2016; Jia et al., 2017). Strigolactone responses require the F-box protein MAX2, which interact with SL receptor DWARF14 (D14) and SMXL proteins, in modulating plant root and shoot development (Felemban et al., 2019). Moreover, MAX2 has been demonstrated to regulate photomorphogenesis, senescence, drought tolerance, and karrikin signaling in Arabidopsis (Bu et al., 2014).

Abscisic acid, a further plant hormone that derives from carotenoids, is a key player in plant response to abiotic stress factors, such as drought and high salinity (Nambara and Marion-Poll, 2005; Peleg and Blumwald, 2011; Finkelstein, 2013). In addition, ABA is an important regulator of seed maturation and dormancy, hypocotyl elongation, and primary root growth (Nambara and Marion-Poll, 2005; Hayashi et al., 2014; Lorrai et al., 2018). The biosynthesis of ABA requires several steps and is initiated by epoxidation of zeaxanthin leading to all-*trans*-violaxanthin, which is catalyzed by the zeaxanthin epoxidase (ZEP; ABA1 in Arabidopsis) (Marin et al., 1996; Xiong et al., 2002; Barrero et al., 2005). All-*trans*-violaxanthin can be converted into all-*trans*-neoxanthin, in a less understood step that involves ABA4/neoxanthin synthase (North et al., 2007). Both all-*trans*-violaxanthin and all-*trans*-neoxanthin can be further converted into their 9-*cis* forms by a not yet identified isomerase. Both 9-*cis*-isomers are the substrate for NCEDs that produce xanthoxin. Xanthoxin is further converted by short-chain dehydrogenase reductase (SDR or ABA2) and abscisic aldehyde oxidase (AAO3), leading to ABA (Supplementary Figure S1; Seo et al., 2000; Cheng et al., 2002; Nambara and Marion-Poll, 2005). *ABA3* is a gene that encodes a molybdenum cofactor that is required for AAO3 enzymatic activity (Bittner et al., 2001).

Recently, we identified zaxinone as a natural apocarotenoid that regulates plant architecture and root growth in rice, likely by repressing SL biosynthesis (Wang et al., 2019). We also showed that zaxinone is formed in rice by the zaxinone synthase (ZAS) that cleaves apo-10′-zeaxanthinal (3-OH-β-apo-10′-carotenal) and represents a member of a sixth CCD subfamily in plants, which is conserved in most land plants but absent in Brassicales species (i.e., *A. thaliana*; Fiorilli et al., 2019; Wang et al., 2019). The loss-of-function mutant of rice *zas* showed decreased content of zaxinone in roots, retarded root and shoot growth and increased SL levels, which could be partially rescued by feeding with zaxinone. Moreover, exogenously applied zaxinone promoted wild-type rice root growth, decreased SL biosynthesis and release, and lowered transcript levels of SL biosynthetic genes under phosphorous (Pi) deficiency (Wang et al., 2019). Nevertheless, zaxinone has been also detected in Arabidopsis, although it lacks ZAS homolog (Mi et al., 2019; Wang et al., 2019). The presence of zaxinone in Arabidopsis and the rice *zas* mutant suggests that this apocarotenoid can be formed via an alternative, ZAS-independent route(s). In this study, we set out to investigate whether zaxinone is a regulatory metabolite in the ZAS-free, non-mycotrophic plant *A. thaliana*.

## Materials and Methods

### Chemicals and Stock Solutions

Zaxinone (all-*trans*-3-OH-apo-13-carotenone) was purchased from Buchem B.V. (Apeldoorn, Netherlands). Strigolactone analog *rac*-GR24 was provided by Prof. Binne Zwanenburg, Radboud University, Netherlands. Abscisic acid was purchased from Sigma chemical company. Hormone stock solutions were prepared in acetone and set at 100, 20, and 20 mM concentration for zaxinone, GR24, and ABA, respectively.

### Plant Growth and Treatment Conditions

Arabidopsis seeds (Col-0), including wild-type and mutant lines; *aba1-6* (CS3772, Barrero et al., 2005), *nced3* (N3KO-6620, Van Norman et al., 2014), *aba2-1* (CS156, González-Guzmán et al., 2002), and *max2-1* (CS9565, received from the laboratory of Prof. O. Leyser, Sainsbury Laboratory, Norwich, United Kingdom) were surface sterilized with 20% bleach for 15 min and cold-treated for 2–3 days at 4°C. For transcripts and metabolite analysis, wild-type seedlings were grown in a hydroponic system adopted from Conn et al. (2013) (Supplementary Figure S2) with a modified half-strength Hoagland nutrient solution. As described before (Abuauf et al., 2018), the nutrient solution consisted of macronutrients in mM: 0.4 K_2_HPO_4_⋅3H_2_O, 0.8 MgSO_4_⋅7H_2_O, 0.18 FeSO_4_⋅7H_2_O, 5.6 NH_4_NO_3_, 0.8 K_2_SO_4_, 0.18 Na_2_EDTA⋅2H_2_O; and micronutrients in μM: 23 H_3_BO_3_, 4.5 MnCl_2_⋅4H_2_O, 1.5 ZnCl_2_, 0.3 CuSO_4_⋅5H_2_O, and 0.1 Na_2_MoO_4_⋅2H_2_O with adjusted pH value of 5.75. Initially, Arabidopsis seedlings were grown in the box system as described by Conn et al. (2013), for 2 weeks. Then, the seedlings were transferred into the 50 ml black tubes and grown another three weeks (see Supplementary Figure S2). Finally, five-week-old plants were treated with different concentrations (0, 0.1, 0.5, 5, 20, and 50 μM) of zaxinone for 6 h. For phosphate starvation, the five-week-old seedlings were kept in a phosphate (K_2_HPO_4_⋅2H_2_O)-free nutrient solution for 3 days. Then, 20 μM of zaxinone and 10 μM of GR24 were used for the treatment. All treatment solutions, including the mock one, contained a final concentration of 0.05% [v/v] acetone. Nutrient solution was replaced twice a week. Seedlings were grown in the growth chamber (Percival) under the following conditions: 22 °C, 10-h-light/14-h-dark, 55% humidity, and 100 μmol m^–2^ s^–1^ light intensity.

### Hypocotyl Elongation Experiment

For the hypocotyl elongation study, wild-type, *aba1-6, nced3*, *aba2-1*, and *max2-1* seeds were germinated on Murashige and Skoog basal salt (MS) medium (1/2 MS salts, 1% agar, 1% sucrose, 0.5 g/l MES, and pH 5.75) for 3 days under the white light condition with a 16-h-light/8-h-dark cycle at 22°C. Subsequently, seedlings were transferred to half-strength Hoagland nutrient agar (1% Agarose, 0.5 g/l MES, pH 5.75) plates containing the broad range concentrations (5, 20, 50, and 100 μM) of zaxinone, and 10 μM of ABA, and GR24. Here, we used half-strength Hoagland nutrient media to maintain the same media condition used in other treatment experiments. All treatment solutions, including the control, contained a final concentration of 0.05% [v/v] acetone. Seedlings were grown vertically under continuous monochromatic red light at 22°C. The monochromatic red light was used to enhance hypocotyl elongation (Jia et al., 2014). After three days, plates were scanned, and hypocotyl length was measured using the ImageJ software.

### Quantitative Real-Time PCR Analysis

Total RNA was extracted from root tissues of Arabidopsis using the TRI-Reagent and Direct-zol RNA MiniPrep Kit (Zymo Research, R2072) according to the manufacturer’s instructions. The concentration and quality of RNA samples were determined using NanoDrop^TM^ 2000 Spectrophotometer. 2 μg of total RNA was used to synthesize total cDNA using iScriptTM cDNA synthesis Kit, following the manufacturer’s protocol (Bio-Rad, 1708890). Quantitative real-time PCR analysis (qRT-PCR) was performed using the ssoAdvancedTM Universal SYBR^®^ Green Supermix, according to the manufacturer’s protocol (Bio-Rad, 10000076382). The mixture was prepared in 96-well plates with a total volume of 20 μl. PCR was performed using the StepOnePlusTM Real-Time PCR System. AtCACS (AT5G46630) gene was employed as an endogenous control. Gene-specific primers used in this study are provided in Supplementary Table S1. The quantitative expression levels of all candidate genes normalized to that of the housekeeping gene *AtCACS* using the equation of 2^–ΔCT^ (Yang et al., 2014).

### Detection and Quantification of MeCLA and ABA

Lyophilized Arabidopsis roots were ground to a fine powder. Approximately 20 mg dry weight (DW) powder was extracted twice with 1 ml of acetone containing 3.3 ng D_6_-5-deoxystrigol (internal standard) for 1 h at 22°C in the dark. After centrifugation, the two extracts were combined and dried under nitrogen. Before UHPLC-MS analysis, the extract was dissolved in 120 μl of acetonitrile:water (50:50, v:v), followed by the filtration with 0.22 μm filter. Detection of MeCLA was performed on UHPLC-Q-Orbitrap-MS (Q-Exactive Plus). Mass Spectrometry parameters were set as follows: capillary temperature of 300°C, AUX gas temperature of 325°C, sheath gas of 45, AUX gas of 20, spray voltage of 4 kV in positive ion mode, and collision energy of 30 eV in PRM analysis. UHPLC separation was performed on a UHPLC (Thermo Scientific^TM^ UltiMate^TM^ 3000 UHPLC) equipped with an ODS column (Waters C_18_, 2.1 × 100 mm, 1.7 μm). The column temperature was maintained at 30°C. The mobile phase consisted of water (solvent A) and methanol (solvent B), both of which contained 0.1% [v/v] formic acid. LC separation was conducted with a linear gradient of 30% B (0 min) to 100% B (16 min) at a flow rate of 0.2 ml/min. For ABA analysis, approximately 5 mg DW powder was extracted twice with 600 μl of 10% methanol containing 1% acetic acid and 1 ng D_6_-ABA for 1 h at 22°C in the dark. After centrifugation, the two extracts were combined and filtered with a 0.22 μm filter for UHPLC-MS analysis. Quantitative analysis of ABA was performed according to Abuauf et al. (2018).

### Strigolactone Collection and *Striga* Seed Germination Bioassays

For *Striga* seed germination bioassays, five-week old Arabidopsis plants grown in hydroponics were treated with 5 μM of zaxinone, and root exudates were collected after 6 h of treatment. The root exudates were run through a C_18_ column, and SLs were eluted with 3 ml acetone as described by Wang et al. (2019). Striga seed germination bioassays were conducted following the procedure described previously (Jamil et al., 2012). Seeds of root parasite *S. hermonthica* were surface sterilized with 50% sodium hypochlorite along with 0.01% Tween-20 for five minutes, followed by six subsequent washings with sterilized Milli-Q water. About 5 mg *Striga* seeds were spread uniformly on a 9 mm glass fiber filter paper disk. Then 12 disks with *Striga* seeds were placed in a 9 cm petri plate, on a sterilized filter paper, moistened with 3 ml Milli-Q water. The petri plates were sealed with parafilm, wrapped in aluminum foil, and put in an incubator at 30°C for pre-conditioning. After 10 days of pre-conditioning, *Striga* seed disks were treated with SL extract (55 μl per disk) collected from root exudates of Arabidopsis, after acetone evaporation in a speed vacuum. GR24 (2.5 μM per disk) and sterile MilliQ water was used as a positive and negative control, respectively. Treated *Striga* seeds were incubated again in the dark for 24 h at 30°C. Germination was recorded under a binocular microscope and used to determine the germination rate (%).

## Results

### Zaxinone Application Increased Transcript Levels of SL Biosynthetic Genes in Arabidopsis

**FIGURE 1:**
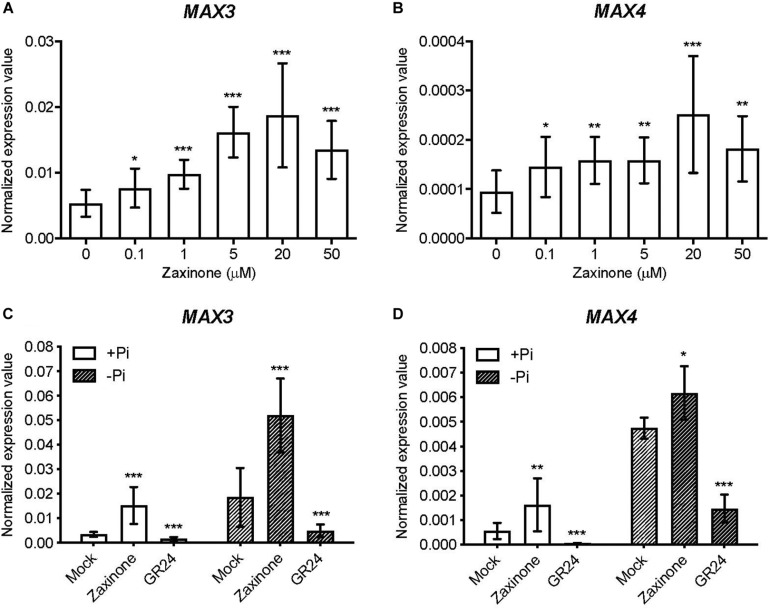
Zaxinone effect on the transcript level of SL biosynthetic genes in Arabidopsis roots. **(A,B)** q-RT-PCR quantification of *MAX3* and *MAX4* transcripts in Arabidopsis roots treated with different zaxinone concentrations for 6 h. **(C,D)** q-RT-PCR quantification of *MAX3* and *MAX4* transcripts in Arabidopsis roots treated with zaxinone (20 μM) or GR24 (2.5 μM) for 6 h under normal Phosphorous (+Pi) and deficient (−Pi) conditions. The quantitative relative expression values of all candidate genes were normalized to that of the housekeeping gene *AtCACS* (AT5G46630) using the equation of 2^–ΔCT^. Values represent at least three biological and two technical replicates. Each biological replicate was combined of two individual plants. Student *t*-test, Mean ± SD, ns, no significant difference; *p < 0.05; **p < 0.01; ***p < 0.001.

To investigate the effect of zaxinone on Arabidopsis SL biosynthesis, we treated hydroponically grown Arabidopsis seedlings, grown under sufficient Pi supply, with different zaxinone concentrations (0, 0.1, 0.5, 5, 20 and 50 μM) for 6 h. Next, we determined the transcript levels of the two SL biosynthetic genes *AtMAX3* (or *AtCCD7*) and *AtMAX4* (or *AtCCD8*) using q-RT-PCR. As shown in Figures 1A,B, the application of zaxinone at all concentrations led to an obvious increase in the mRNA level of both genes. We observed the highest induction upon application of a 20 μM concentration, which led to around 4 and 3 fold increase in transcript levels of *MAX4* and *MAX3*, respectively (Figures 1A,B). Next, we checked whether zaxinone also up-regulates SL biosynthetic genes expression under P starvation that usually triggers SL production (Mayzlish-Gati et al., 2012; Sun et al., 2014). For this purpose, we used a 20 μM concentration of zaxinone. We also applied the SL analog GR24 as a control expected to repress SL biosynthesis via a negative feedback loop (Mashiguchi et al., 2009). As shown in Figures 1C,D, treatment with zaxinone under Pi starvation led to an up-regulation of the transcript level of both *MAX3* and *MAX4*, contrary to the GR24 application. The effect of zaxinone was particularly pronounced on MAX3 (Figures 1C,D). We also checked the impact of zaxinone and GR24 on the expression of other known Arabidopsis SL biosynthetic genes, i.e., *D27*, *MAX1*, and *LBO*, under normal and Pi deficient conditions. Interestingly, we observed a moderate but significant increase of *MAX1* transcripts upon application of both, zaxinone and GR24, under normal conditions, while *D27* and *LBO* were not affected (Supplementary Figure S3). Under Pi deficient conditions, we did not detect significant changes in the mRNA level of *D27*, *MAX1*, and *LBO* (Supplementary Figure S3). Taken together, our results showed that zaxinone positively regulates the transcript level of two key SL biosynthetic genes (*MAX3* and *MAX4*) in Arabidopsis under normal and Pi deficient conditions.

### Zaxinone Enhanced SL Content in Root Tissue and Exudates of Arabidopsis

**FIGURE 2:**
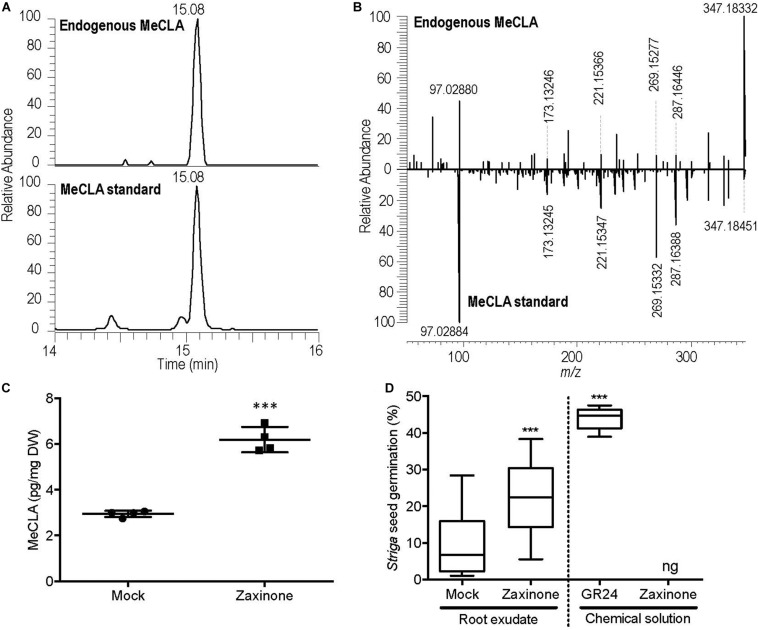
MeCLA quantification in root tissues and measurement of *Striga* seed germinating activity of exudates of Arabidopsis plants after zaxinone treatment. **(A)** EICs of methyl carlactonoate (MeCLA) from Arabidopsis root tissue extract and authentic standard, recorded using UHPLC-MS. **(B)** MS/MS spectra of endogenous MeCLA and MeCLA standard. **(C)** Quantification of MeCLA from Arabidopsis root tissues after 20 μM zaxinone treatment. **(D)** *Striga* seed germination assay conducted by applying root exudates of 5-week-old Arabidopsis plants after 6 h of zaxinone (20 μM) treatment. The Figure shows also the seed germinating activity of solutions containing 2.5 μM of GR24 (positive control) or 20 μM of zaxinone. Student *t*-test, 3–7 biological replicas, Mean ± SD, ns, no significant difference; ng, no germination; *p < 0.05; **p < 0.01; ***p < 0.001.

Since the mRNA levels of two key SL biosynthesis genes, *MAX3* and *MAX4*, were up-regulated in roots by zaxinone application, we further investigated whether this increase leads to a change in SL content in roots and root exudates. For this purpose, we quantified the content of MeCLA, a main active SL reported in Arabidopsis (Abe et al., 2014). Initially, we developed a protocol for the extraction and identification of endogenous MeCLA from root tissue of seedlings grown under normal conditions (Figures 2A,B). Using this protocol, we evaluated the effect of zaxinone treatment on MeCLA content. As shown in Figure 2C, MeCLA content increased about three-fold in Arabidopsis roots, after 6 h zaxinone (20 μM) treatment. Next, we checked whether SL release also increased upon zaxinone application. For this purpose, we conducted a *Striga* seed germination assay with root exudates collected from treated and untreated roots, using GR24 as a positive control. As expected, GR24 solution (2.5 μM) induced *Striga* seed germination to a rate of around 45% (Figure 2D). Exudates collected from zaxinone (20 μM) treated roots (20 μM) showed more than 20% *Striga* seed germination activity, which was around two-fold of that of the control exudates (Figure 2D). To exclude that this increase in germinating activity is caused by zaxinone itself, we also treated *Striga* seeds with this compound. However, we did not detect any effect on the seed germination rate (Figure 2D). Taken jointly, our LC-MS quantification and bioassay data demonstrated that zaxinone positively regulates Arabidopsis SL biosynthesis and release, which is contrary to its effect in rice.

### Zaxinone Triggered ABA Biosynthesis in Arabidopsis

**FIGURE 3:**
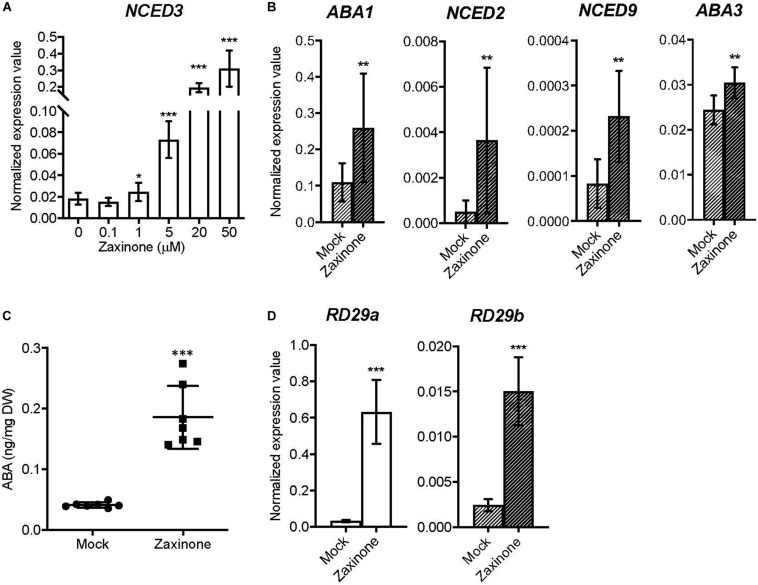
Effect of zaxinone on transcript levels of ABA biosynthetic genes and stress response genes in Arabidopsis roots. **(A)** q-RT-PCR quantification of *NCED3* transcripts in Arabidopsis roots treated with different zaxinone concentrations for 6 h. **(B)** q-RT-PCR quantification of ABA biosynthetic genes (*ABA1*, *NCED2*, and *AAO3*) transcripts in Arabidopsis roots treated with zaxinone (20 μM) for 6 h. **(C)** Quantification of ABA from Arabidopsis root tissues after zaxinone treatment (20 μM). **(D)** Transcripts level of stress response marker genes after 6 h zaxinone (20 μM) treatment. The quantitative relative expression values of all candidate genes were normalized to that of the housekeeping gene *AtCACS* (AT5G46630) using the equation of 2^–ΔCT^. Values represent at least three biological and two technical replicates. Each biological replicate consists of two individual plants. Student *t*-test, Mean ± SD, ns, no significant difference; **p < 0.01; ***p < 0.001.

It was previously shown that the transcript level of SL biosynthetic genes, especially *MAX3* and *MAX4*, can be promoted by abiotic stresses, such as drought, NaCl and Pi deficiency (Cheng et al., 2013; Sun et al., 2014; Saeed et al., 2017; Lv et al., 2018). Therefore, we asked the question whether zaxinone treatment also affects the biosynthesis of ABA, a key regulator of plant’s stress response. To test this, we checked the expression of ABA biosynthesis genes after zaxinone treatment. First of all, we analyzed the expression level of *NCED3*, which encodes the rate-limiting enzyme in the ABA biosynthetic pathway, upon application of different zaxinone concentrations (0, 0.1, 0.5, 5, 20, and 50 μM), using the same hydroponic system. As indicated in Figure 3A, *NCED3* transcript level was induced by zaxinone in a concentration-dependent manner, showing around 1.5, 4, 10, and 15 folds increase upon application of 1, 5, 20, and 50 μM of zaxinone, respectively. However, lower concentrations of zaxinone, i.e., 0.1 μM, did not affect *NCED3* transcript level (Figure 3A). We also analyzed whether the transcript level of other ABA biosynthesis genes, i.e., *ABA1*, *NCEDs* (*NCED2*, *NCED3*, *NCED5*, *NCED6*, and *NCED9*), *ABA2*, *AAO3*, and *ABA3*, are changed after zaxinone treatment. As shown in Figure 3B, zaxinone significantly increased *ABA1*, *NCED2*, *NCED9*, and *ABA3* mRNA levels. While *NCED5*, *NCED6*, *ABA2*, and *AAO3* expression didn’t show a clear change upon zaxinone treatment (Supplementary Figure S4A). In addition, we also investigated the effect of zaxinone on the two common ABA-inducible, stress response genes, *RD29A* (*RESPONSIVE TO DEHYDRATION 29A*) and *RD29B* (*RESPONSIVE TO DEHYDRATION 29B*), and observed a clear induction in the level of the corresponding transcripts (Figure 3D). Next, we quantified ABA content in root and shoot tissues of Arabidopsis plants treated for 6 h with zaxinone (20 μM). This measurement unraveled a four-fold increase in root ABA content (Figure 3C). In contrast, we did not detect any significant change in the level of ABA in shoots (Supplementary Figure S4B).

### Zaxinone Inhibited ABA Dependent Hypocotyl Elongation

**FIGURE 4:**
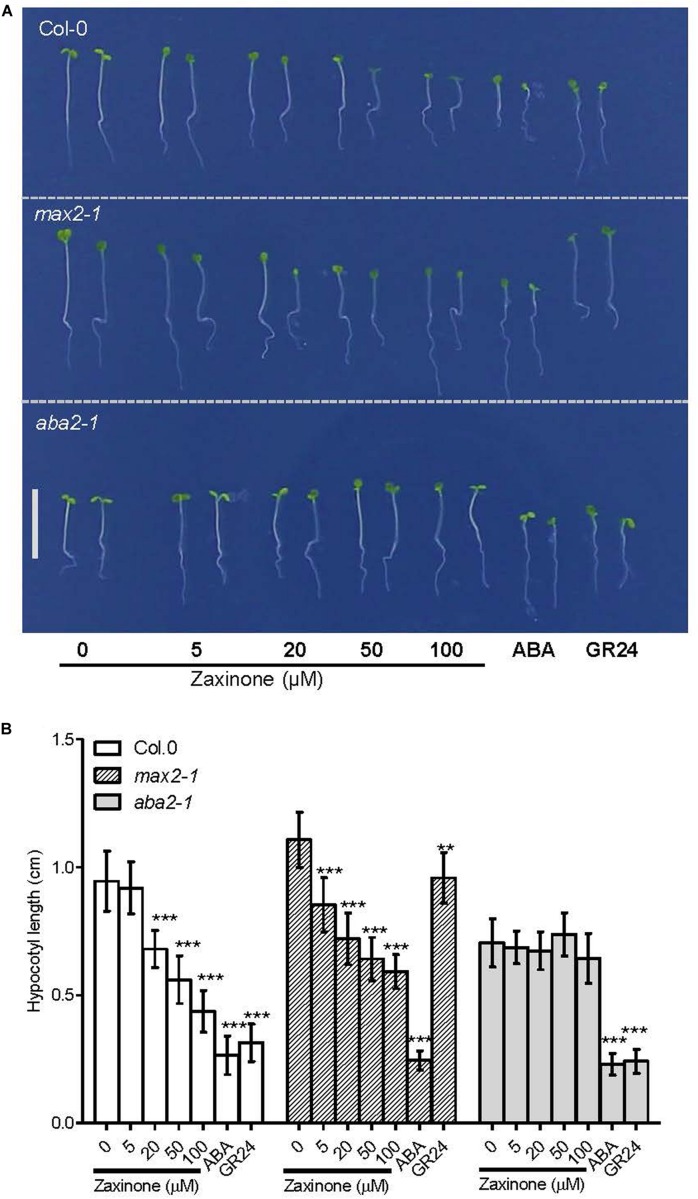
Zaxinone effect on hypocotyl elongation of Arabidopsis wild-type and mutants. **(A)** Effect of different zaxinone concentrations on hypocotyl length of six-day old Arabidopsis Col-0, *max2-1* (SL insensitive), *aba2-1* (ABA deficient) seedlings grown on 1/2 Hoagland Agar plates under continuous red light for three days. Scale bar represents 1 cm. 10 μM of ABA and GR24 were used as a positive control. **(B)** hypocotyl length of the Arabidopsis seedlings in (A). Student *t*-test, 12–16 biological replicas, Mean ± SD, ns, no significant difference; **p < 0.01; ***p < 0.001.

It has been shown that both ABA and GR24 inhibit hypocotyl elongation in several plant species, including Arabidopsis (Hayashi et al., 2014; Jia et al., 2014; Lorrai et al., 2018). Since zaxinone up-regulates both SL and ABA biosynthesis, therefore, we asked the question whether zaxinone can inhibit hypocotyl elongation and, if yes, whether this effect is mediated by ABA or SL increase. To answer this question, we determined the effect of different zaxinone concentrations (5, 20, 50, and 100 μM) on hypocotyl elongation in Col-0 (wild-type), *max2-1* (SL insensitive), and *aba2-1* (ABA deficient) lines grown on 1/2 Hoagland agar under continuous red light exposure, using10 μM of GR24 and ABA as positive controls. Zaxinone treatment significantly inhibited the hypocotyl elongation of wild-type seedlings, at all concentrations except 5 μM. Likewise, hypocotyl elongation of the SL insensitive *max2-1* line was also reduced due to zaxinone treatment hypocotyl elongation was also hindered in all screened concentrations of zaxinone treatment. As shown in Figures 4A,B, the hypocotyl elongation of both genotypes was restrained by zaxinone in a dose-dependent manner. In contrast, hypocotyl elongation of the ABA deficient mutant line *aba2-1* was not affected by broad-range concentration of zaxinone treatment (Figures 4A,B). Similarly, hypocotyl development of another two ABA deficient mutant lines, *aba1-6* and *nced3* were also not hindered by zaxinone (20 μM) application (Supplementary Figures S5A,B). As expected, ABA and GR24 treatment significantly reduced hypocotyl elongation in all lines. These findings reveal that zaxinone negatively regulates hypocotyl elongation and that this effect is mediated by ABA but not SLs.

## Discussion

**FIGURE 5:**
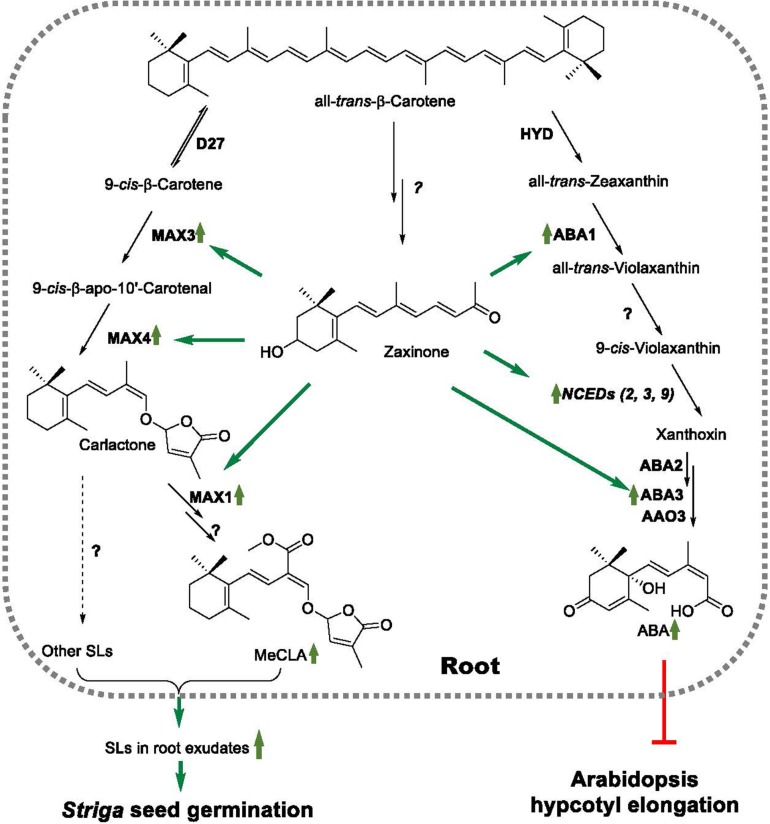
A model of the regulatory activities of zaxinone in Arabidopsis. Zaxinone is an Arabidopsis apocarotenoid metabolite synthesized by an unknown route from b-carotene. Zaxinone increases MeCLA content in Arabidopsis roots by enhancing transcript levels of SL biosynthetic genes, including *MAX3* and *MAX4*, which also results in higher release of SLs and, hence, higher *Striga* seed germinating activity in corresponding root exudates. Zaxinone is also a positive regulator of root ABA biosynthesis, which increases transcript levels of ABA biosynthetic genes (e.g., *ABA1*, and *NCED3*) as well as ABA. Zaxinone effect on ABA content causes also inhibition of hypocotyl elongation.

Zaxinone was recently discovered as a new carotenoid-derived metabolite and growth-promoting compound, which is required for normal growth and development in rice (Wang et al., 2019). Strigolactone quantification and studies performed with SL biosynthesis and perception rice mutants suggested that zaxinone is a negative regulator of SL biosynthesis, which represses levels of SL biosynthetic transcripts under Pi deficient conditions, and that the growth promoting effect of this apocarotenoid likely requires functional SL biosynthesis (Wang et al., 2019). Rice contains ZAS, a member of a sixth CCD subfamily, which contributes to zaxinone production, particularly in roots. However, rice zaxinone is also formed by other ZAS-independent pathways, as demonstrated by the presence of zaxinone in the *zas* mutant (Wang et al., 2019). Interestingly, ZAS orthologs do not exist in genomes of non-AMF host species, including *A. thaliana* (Wang et al., 2019). Furthermore, ZAS genes involvement in the AM symbiosis has been recently discussed by Fiorilli et al. (2019), suggesting that the absence of ZAS ortholog in non-AMF host species may be related to loss of AM symbiosis through their evolution. On the other hand, zaxinone has been also identified in Arabidopsis, although it doesn’t have a close ZAS homolog (Mi et al., 2019; Wang et al., 2019), implying that zaxinone can be generally produced via a route(s) that does not involve ZAS. Here, we investigated whether zaxinone acts as a regulatory metabolite in Arabidopsis, focusing on its possible effect on growth and SL biosynthesis. Our results demonstrate that this apocarotenoid maintains a regulatory role in this ZAS-free, non-AMF species. However, in contrast to rice, zaxinone exerted an inducing effect on SL biosynthesis under normal and Pi deficient conditions. Surprisingly, zaxinone also triggered ABA biosynthesis, which was mirrored by inhibiting hypocotyl growth (Figure 5). The effect of zaxinone on the level of both hormones was mediated by increasing the transcript levels of corresponding biosynthetic genes (Figure 5).

In a first approach, we investigated the effect of the exogenous application on the root growth of Arabidopsis seedlings grown on Agar. As shown in Supplementary Figure S6, we detected inhibition of primary root growth, which we observed at a 5 μM concentration, while treatment with 0.5 μM did not show an effect. To answer the question of whether zaxinone is a regulator of Arabidopsis SL biosynthesis, we first established a hydroponic system that allows an omitting of Pi from the growth medium, a reliable comparison between different growth conditions and the isolation of root exudates. Indeed, the repression of the transcript level of MAX3 and MAX4 upon GR24 treatment suggests that our hydroponics system is appropriate for determining the effect of growth regulators on the expression of SL biosynthetic genes. In addition, we developed an LC-MS based protocol that enabled detection and quantification of MeCLA in Arabidopsis plants grown under normal growth conditions. Our results show clearly that zaxinone is a further apocarotenoid regulatory metabolite, besides the known hormones ABA and SLs and the growth regulator and stress signal cyclocitral (Dickinson et al., 2019; Felemban et al., 2019; Fiorilli et al., 2019; Jia et al., 2019). However, zaxinone application resulted in the increase of *MAX3* and *MAX4* transcript levels, and to a less extent of that of *MAX1*, which indicates that the biological function of zaxinone in Arabidopsis is different from that in rice. In addition, we didn’t observe a significant change of the expression of *D27*, *MAX1*, and *LBO* upon zaxinone application under both Pi sufficient and deficient conditions, except an increase *MAX1* expression after zaxinone treatment under normal conditions. This suggests that different SL biosynthetic genes have distinct responses or sensitivity to zaxinone application. Interestingly, the up-regulation of these transcripts in Arabidopsis was more pronounced under normal, Pi-sufficient conditions, while the effect of zaxinone on rice SL biosynthetic transcripts was only significant under Pi-starvation. The zaxinone-mediated induction of Arabidopsis MAX transcripts led, regardless of Pi-status, to an increase of MeCLA root content and *Striga* seed germinating activity of root exudates, demonstrating that this apocarotenoid is a positive regulator of Arabidopsis SL biosynthesis, which acts at the transcriptional level (Figure 5).

Strigolactones have been implicated in biotic (Decker et al., 2017) and abiotic stress response (Van Ha et al., 2014). Therefore, we tested whether zaxinone can also promote the biosynthesis of the “stress” hormone ABA, assuming that this apocarotenoid metabolite might be involved in Arabidopsis stress response. Our results show that exogenous zaxinone application leads to an increase in the transcript level of ABA biosynthetic genes, including *NCED3* a key gene in ABA formation (Figure 5), and to a striking increase of roots ABA content. Interestingly, we did not observe a change in shoot ABA content, which might be a result of a tissue (root) specificity of the zaxinone effect. In addition to ABA biosynthetic genes, we also observed an induction of *RD29a* and *RD29b* transcripts that are known to respond to stress conditions (Yamaguchi-Shinozaki and Shinozaki, 1993; Msanne et al., 2011).

Taken together, our study suggests that zaxinone is a regulatory metabolite in Arabidopsis roots, which enhances the level of ABA and SLs. This effect indicates that zaxinone may act as a stress signal in Arabidopsis roots, rather than being a growth-promoting compound as observed in rice. Indeed, zaxinone is the first signaling molecule/regulatory metabolite reported to simultaneously induce the biosynthesis of these two hormones. The question about the synthesis of this apocarotenoid in ZAS-free plants, such as Arabidopsis, remains elusive and is the subject of a future work.

## Data Availability Statement

All datasets generated for this study are included in the article/Supplementary Material.

## Author Contributions

SA-B and AA conceived and designed the research. AA performed gene expression analysis and prepared the plant materials. JM detected and quantified the metabolites. MJ conducted *Striga* seed germination bioassays. K-PJ assisted to perform the agar plate experiment. AA and JM analyzed the data and generated the figures. QF performed SL collection from root exudates. SA-B edited and approved the manuscript. All authors contributed for manuscript writing and respective parts.

## Conflict of Interest

The authors declare that the research was conducted in the absence of any commercial or financial relationships that could be construed as a potential conflict of interest.

## Supplementary Material

FIGURE S1 Schematic biosynthetic pathway for SL and ABA in *Arabidopsis thaliana*. Left, functionally characterized enzymes involved in SL biosynthesis in *Arabidopsis thaliana*: D27, DWARF27; CCDs, carotenoid cleavage dioxygenases; MAXs; more axillary growth. Right, functionally characterized enzymes involved in ABA biosynthesis in *Arabidopsis thaliana*: HYD, carotene β-ionone ring hydroxylase; ABA1/ZEP, zeaxanthin epoxidase NCEDs, 9-*cis*-epoxycarotenoid dioxygenases; ABA2/SDR, short-chain alcohol dehydrogenase/reductase; AAO3, abscisic aldehyde oxidase, ABA3, molybdenum cofactor; ?, unknown enzyme.

FIGURE S2 Hydroponics system set-up for growing plants and treatment. Initially, Arabidopsis seedlings were grown in the box system as described by Conn et al., 2013 for two weeks. Seedlings were then transferred into 50 ml black tubes and grown for another three weeks under the following conditions: 22°C, 10 h day/14 h dark, 100 μmol m^–2^ s^–1^, 1/2 Hoagland nutrient solution were used and replaced twice a week. Blue, red, and orange lines represent normal, Pi starvation, and zaxinone treatments, respectively. HydroLogix Pipet Tips box used to grow the seedlings.

FIGURE S3 Zaxinone effect on transcript level of SL biosynthetic genes in five- weeks-old Arabidopsis root tissue under normal phosphorous (+ Pi) and deficient (−Pi) conditions. (**(A–C)** *D27*, *MAX1*, and *LBO* transcript levels under normal phosphorous (+ Pi) and deficient (−Pi) condition after 6 hr of 20 μM of zaxinone treatment. The quantitative relative expression values of all candidate genes were normalized to that of the housekeeping gene *AtCACS* (AT5G46630) using the equation of 2^–ΔCT^. Values represent at least three biological and two technical replicates. Each biological replicate was the pool of two individual plants. Student *t*-test, Mean ± SD, ns, no significant difference, ^∗^*p* < 0.05.

FIGURE S4 Zaxinone effect on transcript levels of ABA biosynthetic genes in root tissues and ABA content in shoot tissues of five-weeks-old Arabidopsis. **(A)** ABA biosynthetic genes, including *NCED5*, *NCED6*, *ABA2*, and *AAO3* transcripts level after 6 h of zaxinone (20 μM) treatment. The quantitative relative expression values of all candidate genes were normalized to that of the housekeeping gene *AtCACS* (AT5G46630) using the equation of 2^–ΔCT^. Each biological replicate was combined of two individual plants. **(B)** Quantification of ABA from Arabidopsis shoot tissues after treatment with zaxinone (20 μM). For quantification, seven biological replicates were used, and two individual plants were combined as a single replicate. Student *t*-test, Mean ± SD, ns, no significant difference, ^∗∗^*p* < 0.01; ^∗∗∗^*p* < 0.001.

FIGURE S5 Hypocotyl Elongation of Wild-type (Col-0) and ABA deficient mutants (*aba1-6* and *nced3)* in response to zaxinone, GR24 and ABA. **(A)** Six-day-old Arabidopsis Col-0, *aba1-6*, and *nced3* seedlings grown on 1/2 Hoagland Agar plates with zaxinone (20 μM), GR24 (10 μM) and ABA (10 μM) under continuous red light for three days. **(B)** The column graph represents the Hypocotyl length of Six-day-old Arabidopsis Col-0, *aba1-6, nced3* seedlings grown on 1/2 Hoagland Agar plates with zaxinone (20 μM), GR24 (10 μM) and ABA (10 μM) under continuous red light for three days. ABA and GR24 treated as positive control, respectively. Student t-test, 12-16 biological replicas, Mean ± SD, ns; no significant difference, ^∗∗∗^*p* < 0.001.

FIGURE S6 Zaxinone effect on primary root growth of Arabidopsis. **(A)** Arabidopsis (Col-0) seedlings were grown for one week on 1/2 MS with 1% Sucrose Agar plate and then transferred to 1/2 Hoagland Agar plates with different concentrations (0, 0.5 and 5 μM) of zaxinone treatment and kept for five days. **(B)** Primary root length of 12-day-old Arabidopsis (Col-0) seedlings after five days treatment with different zaxinone concentrations. The seedlings were grown under white light with a 16-h-light/8-h-dark cycle at 22°C. Student *t*-test, 12-16 biological replicates, Mean ± SD, ns, no significant difference; ^∗∗∗^*p* < 0.001.

TABLE S1 Sequence of gene-specific primers used for qRT-PCR analysis.
