# Supplementary material for: The Apocarotenoid Zaxinone Is a Positive Regulator of Strigolactone and Abscisic Acid Biosynthesis in Arabidopsis Roots
Source: Front Plant Sci. 2020 May 14;11:578. doi: 10.3389/fpls.2020.00578 (PMC7240130; doi:10.3389/fpls.2020.00578)

## Slide 1
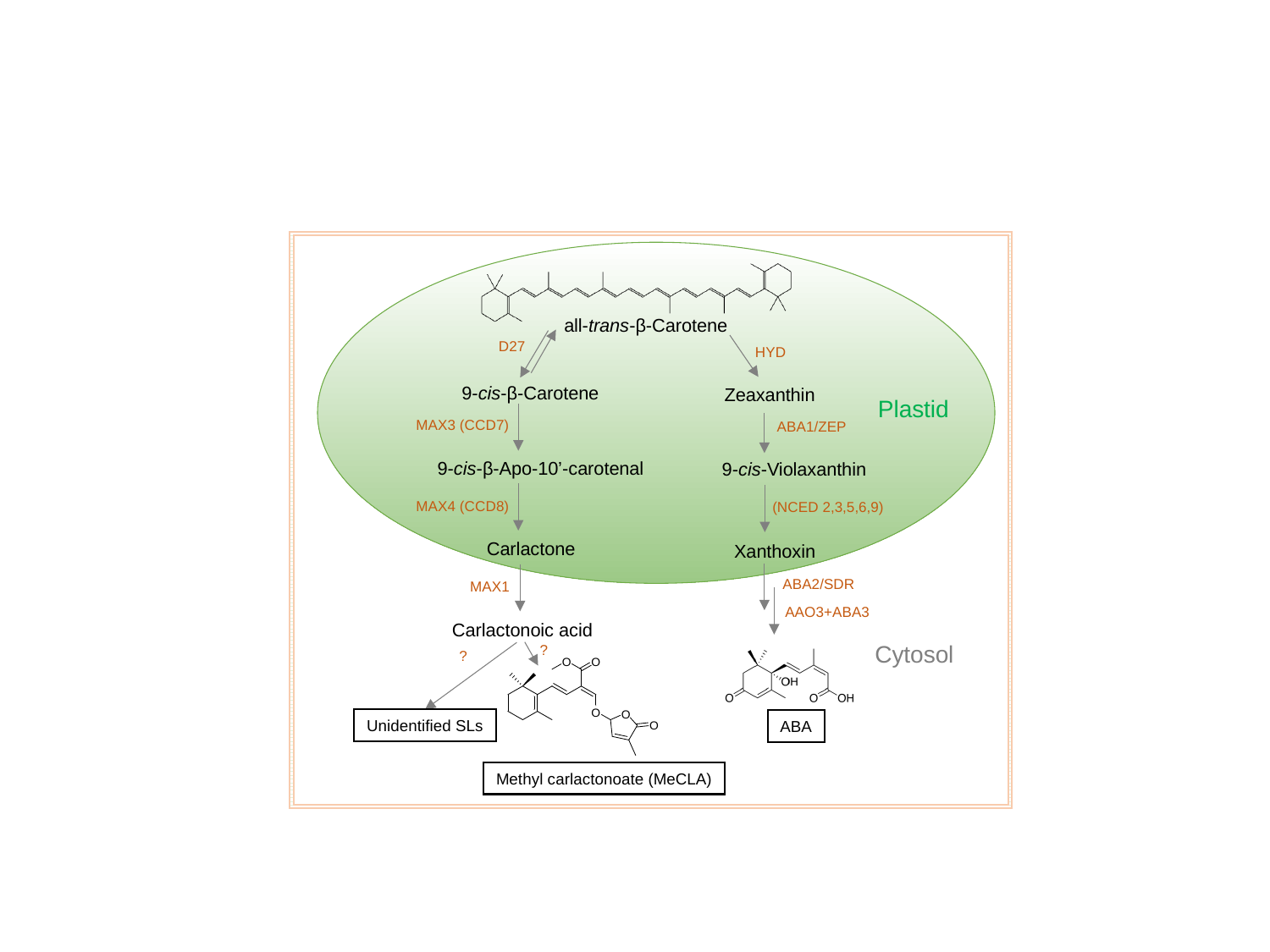

all-trans-β-Carotene
D27
HYD
9-cis-β-Carotene
Zeaxanthin
Plastid
MAX3 (CCD7)
ABA1/ZEP
9-cis-β-Apo-10’-carotenal
9-cis-Violaxanthin
MAX4 (CCD8)
(NCED 2,3,5,6,9)
Carlactone
Xanthoxin
ABA2/SDR
MAX1
AAO3+ABA3
Carlactonoic acid
Cytosol
?
Unidentified SLs
ABA
Methyl carlactonoate (MeCLA)
?

## Slide 2
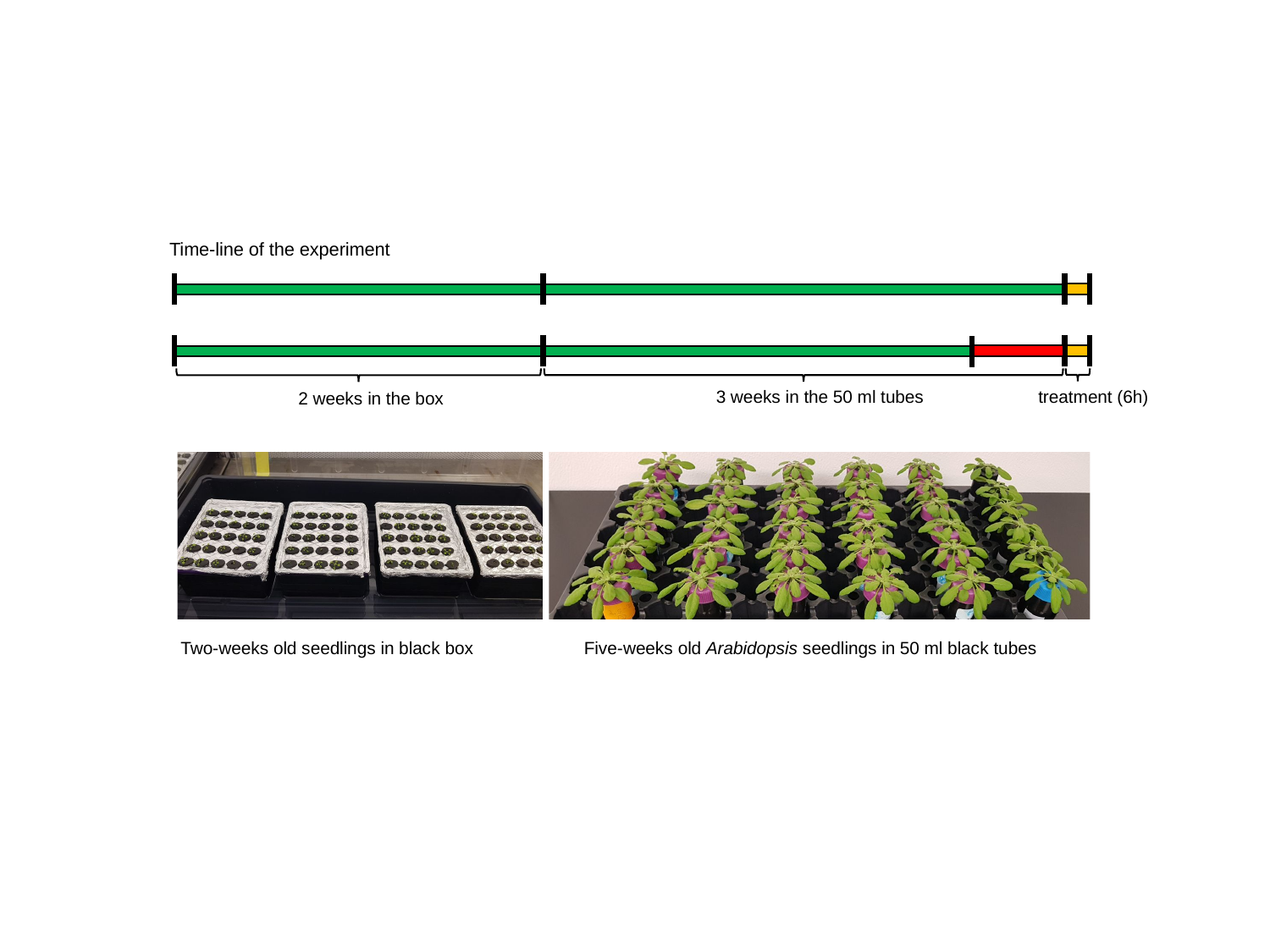

Time-line of the experiment
treatment (6h)
3 weeks in the 50 ml tubes
2 weeks in the box
Two-weeks old seedlings in black box
Five-weeks old Arabidopsis seedlings in 50 ml black tubes

## Slide 3
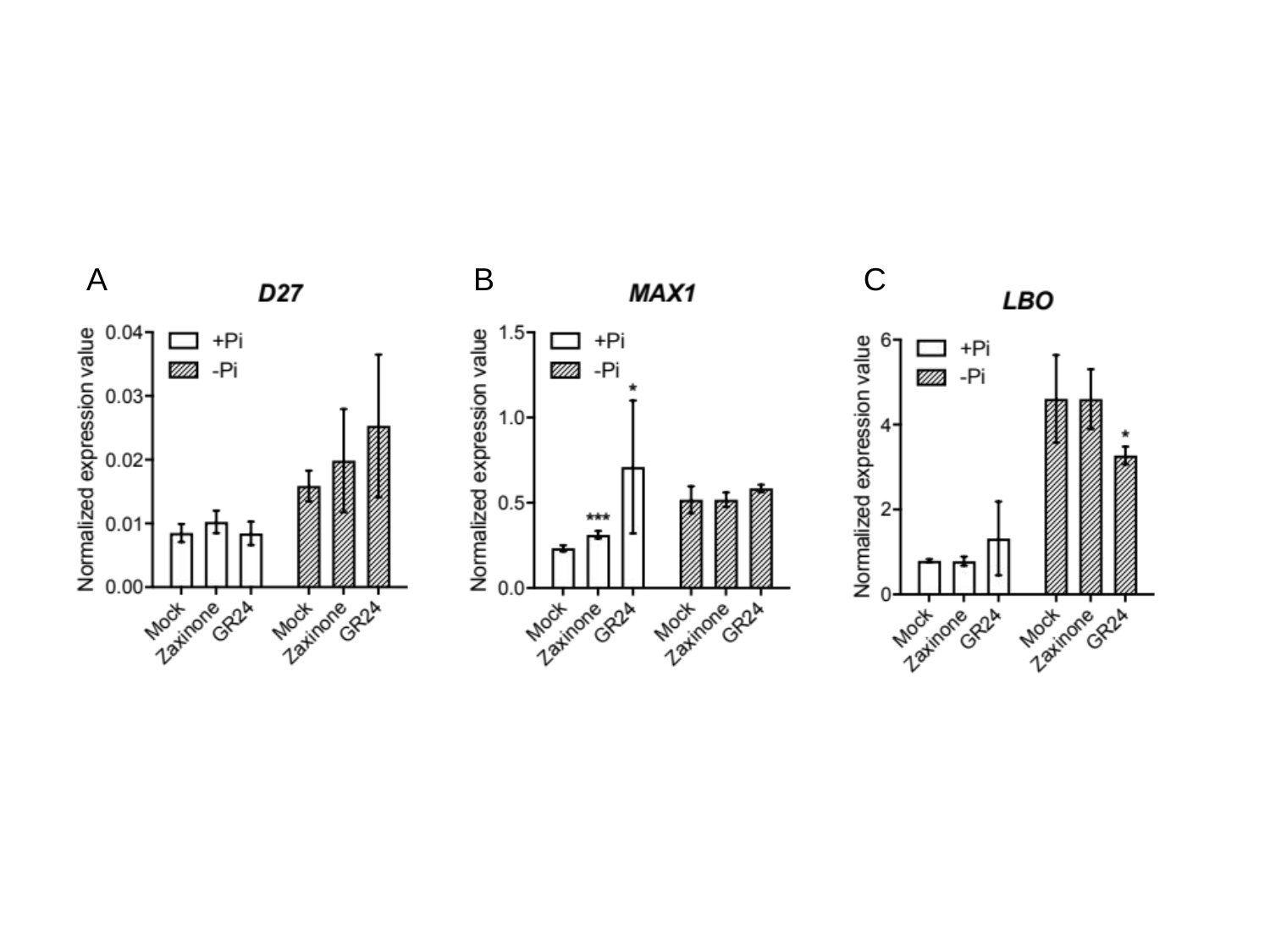

A
 B
 C

## Slide 4
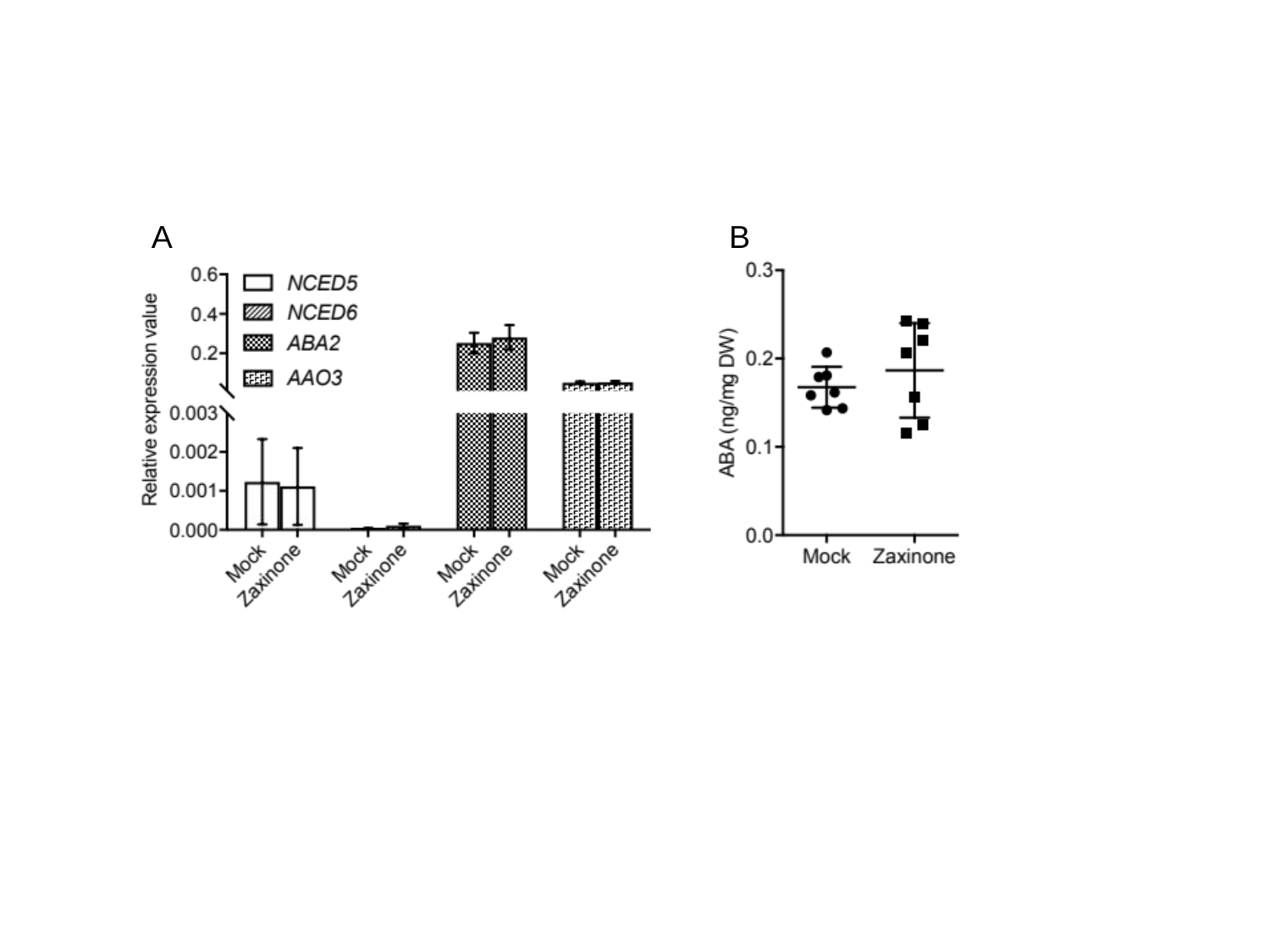

A
 B

## Slide 5
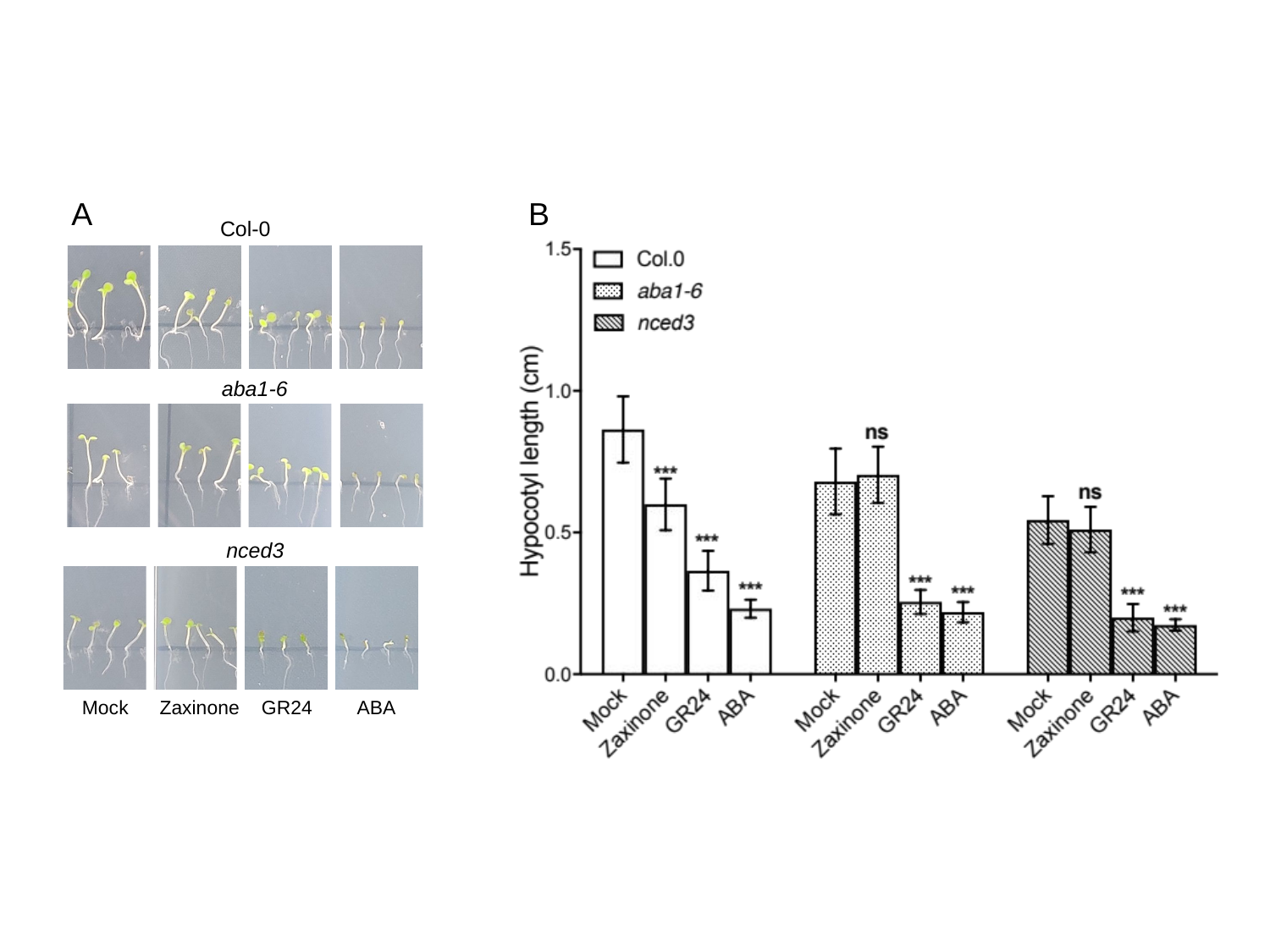

A
 B
Col-0
aba1-6
nced3
GR24
ABA
Mock
Zaxinone

## Slide 6
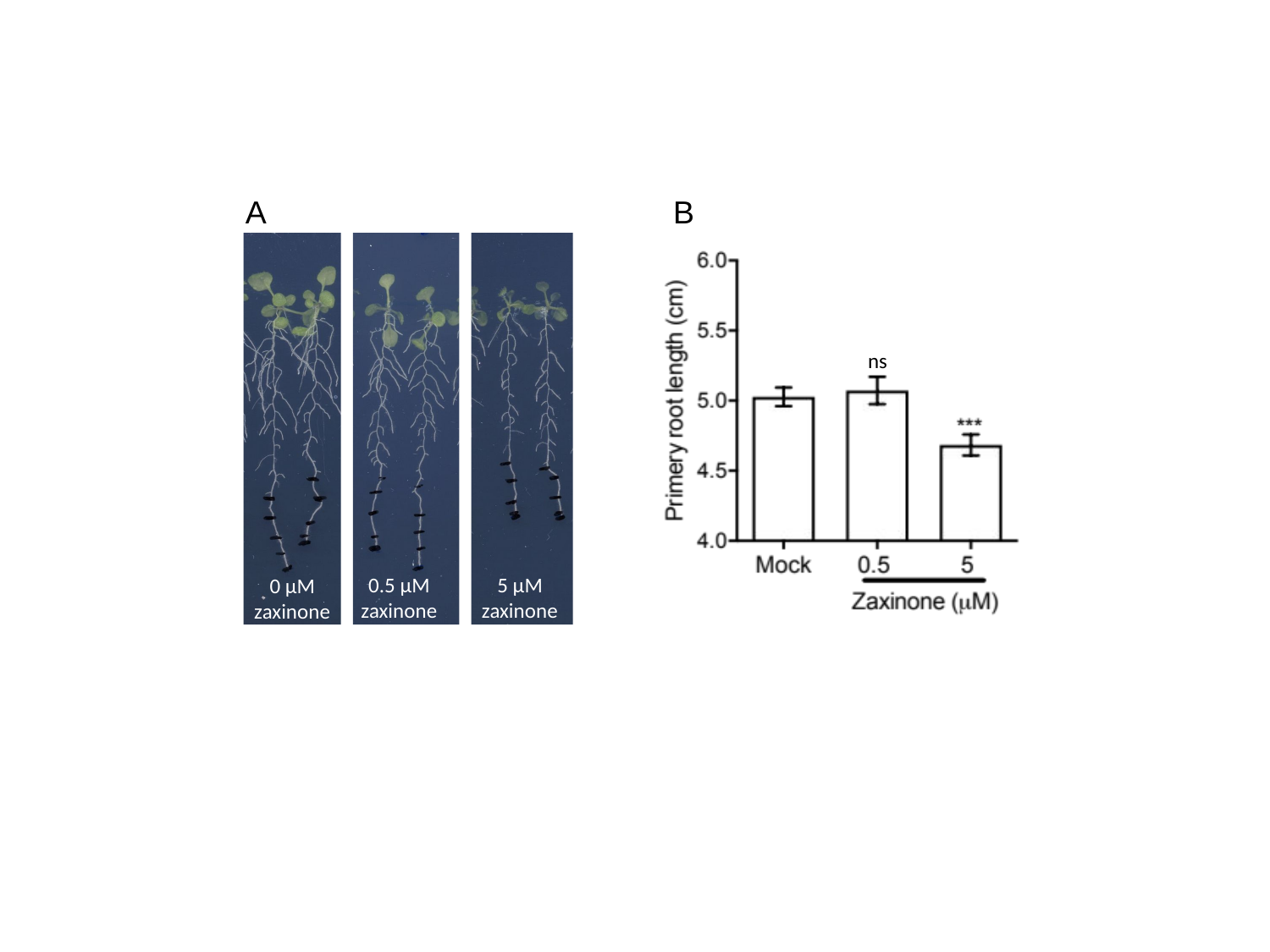

A
 B
0.5 μM zaxinone
5 μM zaxinone
ns
0 μM zaxinone

## Slide 7
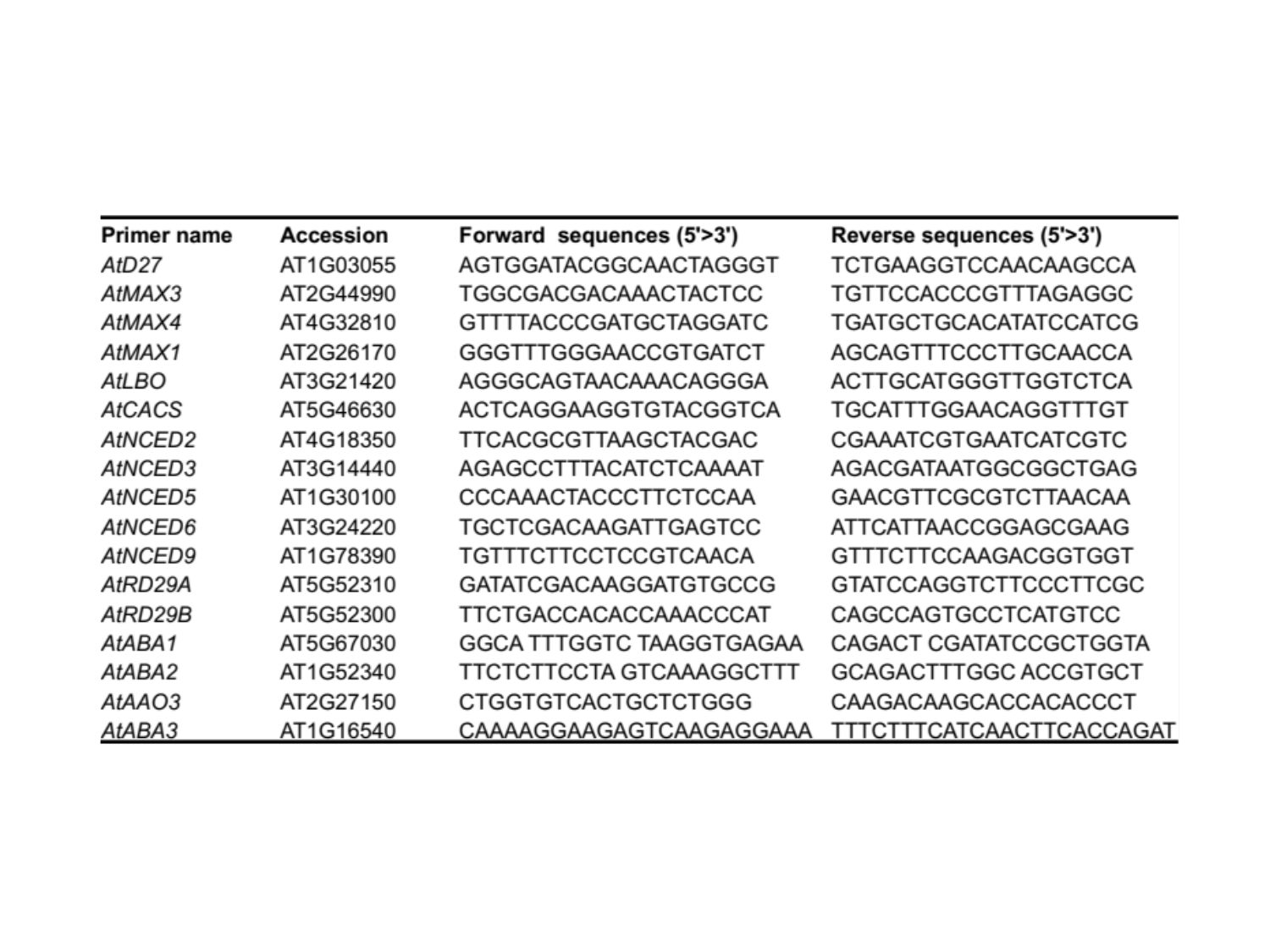

Supplement: FIGURE S1 — Schematic biosynthetic pathway for SL and ABA in Arabidopsis thaliana. Left, functionally characterized enzymes involved in SL biosynthesis in Arabidopsis thaliana: D27, DWARF27; CCDs, carotenoid cleavage dioxygenases; MAXs; more axillary growth. Right, functionally characterized enzymes involved in ABA biosynthesis in Arabidopsis thaliana: HYD, carotene β-ionone ring hydroxylase; ABA1/ZEP, zeaxanthin epoxidase NCEDs, 9-cis-epoxycarotenoid dioxygenases; ABA2/SDR, short-chain alcohol dehydrogenase/reductase; AAO3, abscisic aldehyde oxidase, ABA3, molybdenum cofactor; ?, unknown enzyme. [file Presentation_1.PPTX]
